# Metachronous renal vein and artery injure after percutaneous nephrostolithotomy

**DOI:** 10.1186/1471-2490-13-69

**Published:** 2013-12-05

**Authors:** Chaojun Wang, Shanwen Chen, Fuqing Tang, Baihua Shen

**Affiliations:** 1 Department of Urology, the First Affiliated Hospital, Medical of College, Zhejiang University, No. 79 Qing Chun road, Hangzhou 310003, China

**Keywords:** Percutaneous nephrostolithotomy, Misplacement, Pseudoaneurysm, Computed tomography angiography

## Abstract

**Background:**

Percutaneous nephrostolithotomy is important approach for kidney stones removal. A percutaneous nephrostomy drainage tube placement is an effective method to stop venous bleeding. Occasionally, the catheter can pierce into the renal parenchyma, and migrate into the renal vein even to the vena cava.

**Case presentation:**

A 66-year-old woman underwent a percutaneous nephrostolithotomy for kidney staghorn stone complicating severe bleeding. A computed tomography angiography showed the percutaneous nephrostomy drainage tube inside the renal vein. The percutaneous nephrostomy drainage tube was withdrawn 3 cm back to the renal parenchyma/sinus/pelvis in stages with the surgical team on standby. Seven days later, the patient developed severe hematuria. Computed tomography angiography demonstrated the pseudoaneurysm located near the percutaneous nephrostomy drainage tube. Pseudoaneurysm is embolized successfully.

**Conclusion:**

Our case shows intravenous misplacement of the nephrostomy tube and subsequent pseudoaneurysm after percutaneous nephrostolithotomy. To our knowledge, this seems to be the first documentation of major bleeding from the injury to both renal vein and artery. The percutaneous nephrostomy drainage tube can be withdrawn back to the renal parenchyma/sinus/pelvis in stages with the surgical team on standby, and the withdrawn distance may vary according to patient and catheter position.

## Background

Percutaneous nephrostolithotomy (PCNL) was introduced by Fernström and Johansson in 1976 [1], and it has remained as important approach for kidney stones removal since its inception. Venous bleeding during percutaneous procedures is mild and resolves spontaneously or responds to simple maneuvers such as placement a large caliber nephrostomy tube into the tract [2]. Severe bleeding-complications of percutaneous renal surgery are commonly arterial in nature [3,4]. Hemorrhage caused by pseudoaneurysms usually occurs in the postoperative period and can be managed best by selective angiographic embolization. Occasionally nephrectomy is required for refractory bleeding. To our knowledge, major bleeding from injury to both the renal vein and artery has not been reported. We report one case of intravenous misplacement of the nephrostomy tube and subsequent pseudoaneurysms following percutaneous nephrostolithotomy.

## Case presentation

A 66-year-old woman underwent a PCNL for a staghorn stone in the left kidney. Access to the excretory system was achieved by fascial dilators, and a safety guide wire was used during the procedure. An ultrasonic energy source was used to shatter the stone. Severe venous bleeding was noted during the fragmentation process. The procedure was interrupted; a percutaneous nephrostomy drainage tube (PNDT) was inserted and closed in order to control bleeding within the excretory system.

PNDT was reopened on the second postoperative day, and intense bleeding was observed through the drainage tube, which was immediately closed. The patient presented with normal blood pressure (128/79 mmHg) and a decreased hemoglobin (7.3 g/dL). The patient was transfused with four units of blood, and she remained hemodinamically stable.

**Figure 1:**
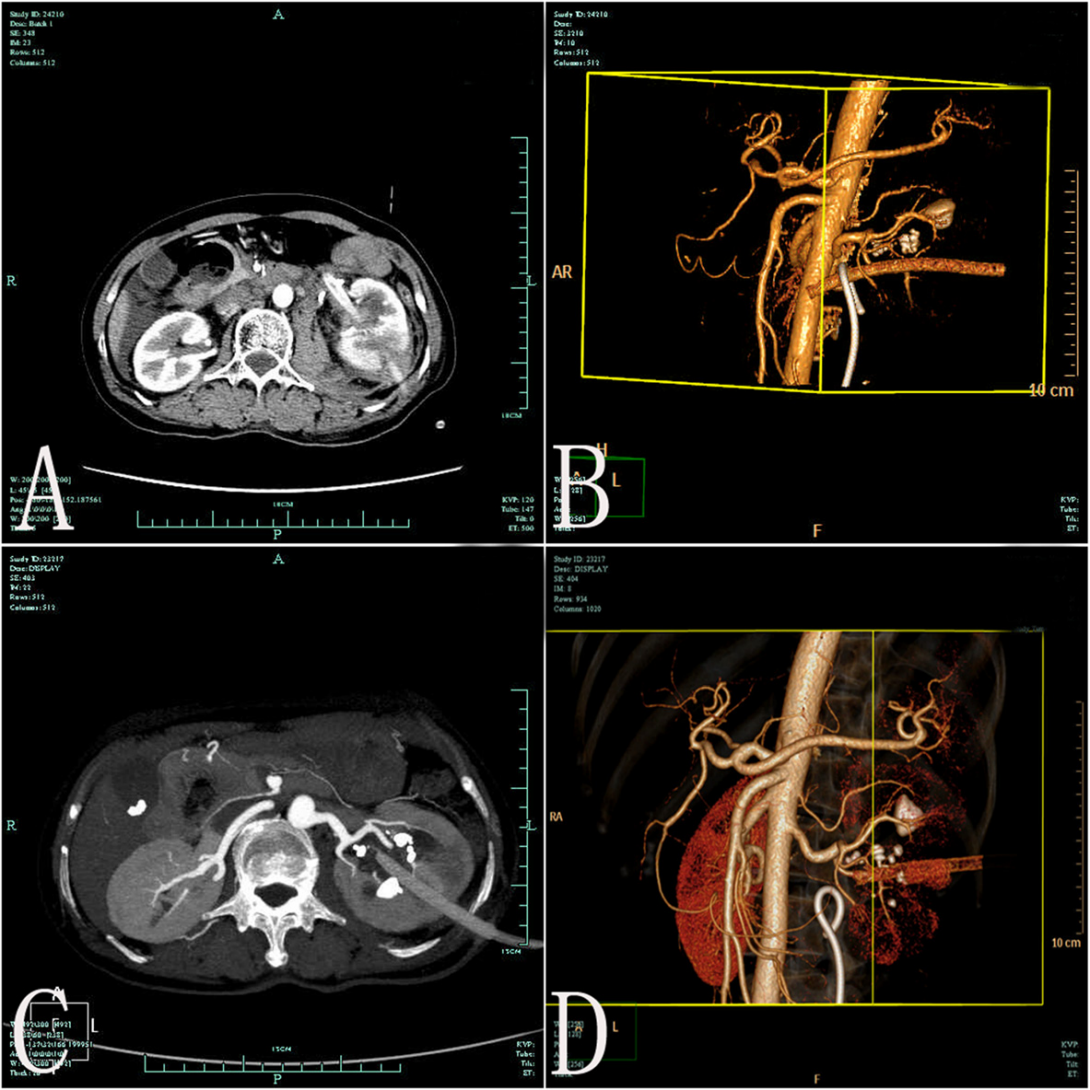
**Axial contrast enhanced CT and volume rendering image of CT angiography. A-B)** Axial contrast enhanced CT and volume rendering image of CT angiography demonstrate percutaneous nephrostomy drainage tube inside the left renal vein; **C-D)** Axial contrast enhanced CT and volume rendering image of CT angiography reveal percutaneous nephrostomy drainage tube inside the left renal pelvis after the percutaneous nephrostomy drainage tube was withdrawn in stages.

On the second post-operative day, a computed tomography angiography showed the PNDT in the left renal vein (Figure 1). Vascular flow was monitored through regular Doppler ultrasonography. Doppler ultrasound showed that there was no renal vein or vena cava thrombosis. The patient was taken to the operating room, and the PNDT was withdrawn 3 cm back to the renal sinus/parenchyma under fluoroscopy control (the contrast material through the catheter when we withdrew the catheter) with the surgical team on standby ready to intervene. 24 hours later the PNDT was repositioned in the collecting system under fluoroscopy through the same way. Bleeding of the PNDT immediately slowed down and the urine became clear during the next day. The patient was discharged on the sixth post-operative day with a hemoglobin level of 9.3 g/dL.

**Figure 2:**
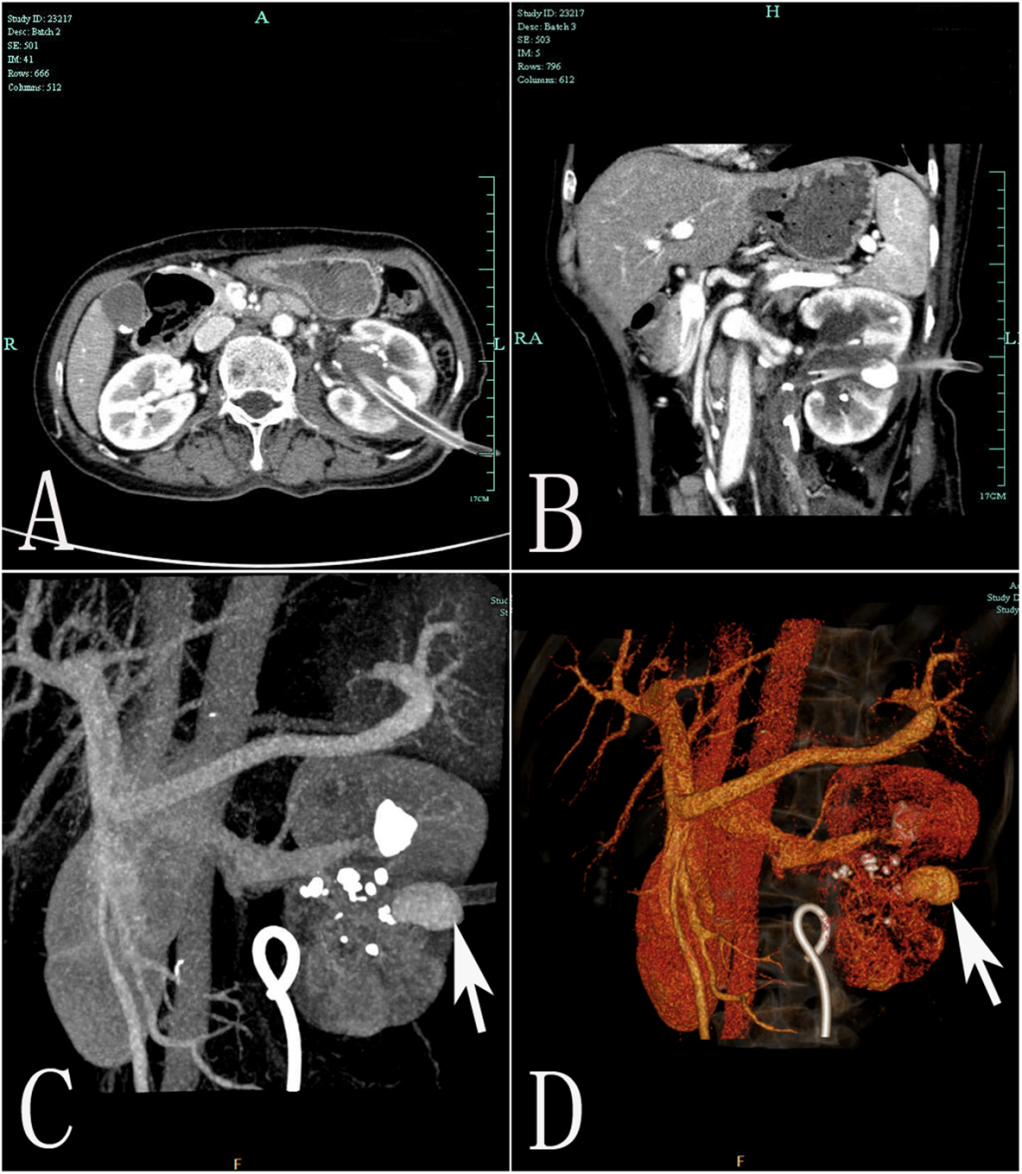
**Imaging of CT. A)** Axial contrast enhanced CT demonstrates the pseudoaneurysm (an oval structure) arising from the lower pole of the left kidney, the pseudoaneurysm was located near the percutaneous nephrostomy tract. **B-D)** Coronal reformatted **(B)**, maximum intensity projection **(C)** and volume rendering **(D)** images of CT angiography reveal the pseudoaneurysm (arrow) arising from the lower polar segmental artery.

**Figure 3:**
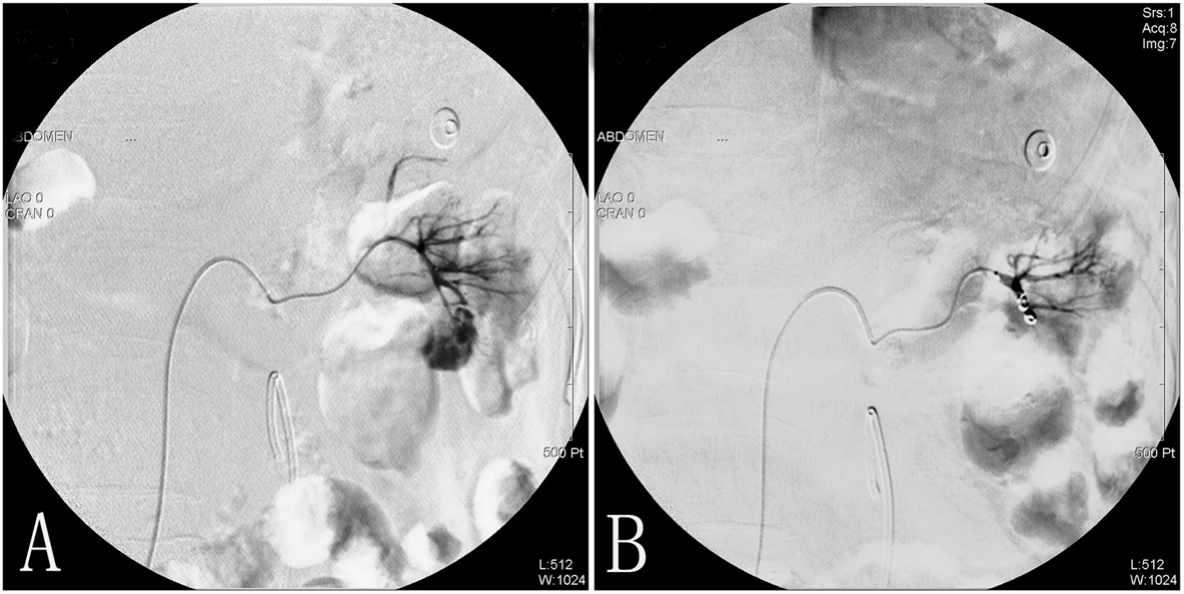
**Angiographic evidence of renal artery pseudoaneurysm before and after angioembolization with coils. A)** Arteriography demonstrates one classic pseudaneursym (arrow) “blush” arising from the lower polar segmental artery of left renal artery. **B)** Pseudoaneurysm is embolized selectively with two metal coils (arrow). No further contrast medium leakage can be seen following embolisation.

On the eighth post-operative day, the patient developed gross hematuria and severe pain of the left loin. A significant drop of the hemoglobin level was noted (from 9.3 g/dL to 6.8 g/dL) but she remained hemodynamically stable and the coagulation parameters were within normal limits. An abdominal computed tomography scan revealed a large left perinephric hematoma. She was initially treated conservatively with bed rest and transfusions but gross hematuria persisted. After failed resuscitation, a computed tomography angiography was arranged, which revealed a pseudoaneurysm in the left renal artery (Figure 2). A selective left renal angiogram was arranged in order to achieve endovascular control of the bleeding vessel. Active bleeding from the pseudoaneurysm at a branch of the left renal artery with contrast extravasation was shown (Figure 3). The bleeding was embolized selectively with two 2 mm*2 cm metal coils. Twenty-four hours later, hematuria ceased and the patient remained hemodynamically stable.

Following above procedure, there was no further gross hematuria. On the thirtieth post-operative day, a nephrostogram revealed the tract had sealed completely, and the PNDT was removed uneventfully. After an 18-month follow up, her post-operative hemoglobin and serum creatinine remained normal.

## Discussion

A PNDT placement in the collecting system following PCNL is a routine practice. It is an effective method to stop venous bleeding [5]. Occasionally, the catheter can pierce into the renal parenchyma, and migrate into the renal vein even to the vena cava [5]. The proximity of the renal vein to the renal pelvis and major posterior calices predisposes them to injury during PCNL [6]. Our patient had complex staghorn renal calculi involving the renal pelvis. Concomitant infection and inflammation may have made the renal pelvic wall more friable and susceptible to the injury.

The mechanism of injury was different in resulting from dilating too medially, overzealous tract dilation, injury to a caliceal infundibulum, and pushing too hard on a renal pelvic stone with the ultrasound lithotrite probe as in our patient. Understanding since the renal pelvis is a delicate structure with a thin muscular layer and there is no structural support for it, special care should be taken to stones in the pelvis or lodged at the ureteropelvic junction. By using the ultrasound probe gingerly with firm but gentle intermittent pressure against the stone and without jerky to and fro motions can prevent the stone or the instrument from perforating the collecting system.

Abdallah Geara presented a case of visualization of the renal vein during pyelography after nephrostomy, and examination by microscopy showed the presence of tears in the fornix of the pelvic cavity that extend into the kidney parenchyma [7]. However, significant venous injuries during percutaneous renal surgery probably are under diagnosed [8]. Since contrast material generally is not injected during percutaneous procedures to prevent extravasation from obscuring the fluoroscopic image, major injuries may be missed. Clotting of these venous injuries may occur during the operation and, therefore, the injury may not be evident on nephrostograms performed at the conclusion of the procedure or at follow-up.

In cases where misplacement of the tube is detected, depending on the postoperative time elapsed, relocation of the nephrostomy tube under fluoroscopy is strongly recommended, and the surgical team must stand ready to operate in case an open emergency procedure is required [9]. Our case illustrates that control on the hemorrhage could be achieved with the catheter being withdrawn in stages, and laparotomy could be avoided despite of the perforation into the major renal vein. If the hemorrhage could not be controlled, in Gupta M’experience, the tamponading nephrostomy catheter is optimally suited for this purpose [8]. Holding mild tension on the catheter against the site of injury, the balloon can be further inflated if necessary until extravasation into the vein ceases. However, those cases involved prolonged balloon inflation and risked pressure necrosis. Our case highlights the importance of prompt diagnosis of renal-vein perforation and demonstrates that this can be managed conservatively by the catheter being withdrawn in stages or a tamponading nephrostomy catheter.

This study is the fifth report in the literature regarding misplacement of a nephrostomy tube into the vascular system [8-10], yet, it is the first report under the guide of ultrasound, and is also the first report with such a complication of vein and artery following PCNL.

It is an uncommon case of renal artery pseudoaneurysm because of its delayed occurrence after invasive procedure. The percutaneous tract disrupts the normal vessel wall and a pseudoaneurysm is formed from the tissues surrounding the high-pressure arterial system, resulting in recanalisation between the intravascular and extravascular space that produces a pulsating, encapsulated hematoma. The vascular compromise is initially controlled by a combination of decreased blood flow, artery spasm, coagulation, and a tamponading effect of the surrounding tissue, which initially prevents hemorrhage and leads to its nonrecognition in the operating room. As these temporizing measures begin to degrade, the patient becomes more active, the blood flow to this vessel increases and the pseudoaneurysm may eventually grow and becomes unstable. With erosion into the pelvicaliceal system, gross hematuria thus occurs. Post-PCNL pseudoaneurysm is usually located in the peripheral arteries. This might explain the absence of significant bleeding intraoperatively and postoperatively. This is supported by the fact that our patient angiography identified that the pseudoaneurysm was located near the punctured cavity.

The natural history of pseudoaneurysm ranges from spontaneous resolution to acute rupture. By far, the most common presentation is gross hematuria. Patients are often clinically stable and can present with nonspecific symptoms including flank pain, hypertension, arterial bruits, deterioration of renal function and worsening lower urinary tract symptoms. Patients usually present in the first few weeks (ranging from 1 day to 5 months) postoperatively.

The diagnosis of intrarenal pseudoaneurysm is challenging. Clinical diagnosis can be done through invasive (angiography) and non-invasive (angio CT or Doppler ultrasonography) methods. Arteriography remains the gold standard diagnostic tool, although multiplanar contrast enhanced computed tomography is suitable alternatives. The advantages of angiography in this setting include high sensitivity in identifying the pseudoaneurysm (which usually appears as a round or oval structure arising from the main renal artery or one of its branches) and the potential to achieve simultaneous endovascular management of these lesions, with success rates exceeding 90%.10 Superselective embolisation is highly efficient in achieving pseudoaneurysm occlusion through the injection of a permanent agent at the fistulous point. Materials such as ethanol, metal coil, gel foam particles and N-butyl-2-cyanoacrylate have been successfully used for embolisation [11,12]. No significant difference was found between the preangioembolization and postangioembolization serum creatinine, similar to that reported by Martin et al. [11]. Pseudoaneurysm appears as a focal area of high attenuation with a similar enhancement to the adjacent vessels. The advantage of computed tomography imaging is that these modalities not only can diagnose pseudoaneurysms but can also detect other possible intra-abdominal pathologies that may be responsible for the presenting symptoms. A three-dimensional reconstruction can be useful for differentiating a pseudoaneurysm from urinoma, which may, at times, be difficult to distinguish from each other.

## Conclusions

Our case shows intravenous misplacement of the nephrostomy tube and subsequent pseudoaneurysm after percutaneous nephrostolithotomy. To our knowledge, this seems to be the first documentation of major bleeding from the injury to both renal vein and artery. The percutaneous nephrostomy drainage tube can be withdrawn 3 cm back to the renal parenchyma/sinus/pelvis in stages with the surgical team on standby, and the withdrawn distance may vary according to patient and catheter position.

## Consent

Written informed consent was obtained from the patient for publication of this manuscript and accompanying images. A copy of the written consent is available for review by the Editor-in-Chief of this journal.

## Abbreviations

PCNL: Percutaneous nephrostolithotomy; PNDT: Percutaneous nephrostomy drainage tube.

## Competing interests

The authors declare that they have no competing interests.

## Authors’ contributions

CW and FT cared for the patient and drafted the report. BS cared for the patient. SC revised and approved the final version of the manuscript. All authors reviewed the report and approved the final version of the manuscript.

## Pre-publication history

The pre-publication history for this paper can be accessed here:

http://www.biomedcentral.com/1471-2490/13/69/prepub
